# Targeting CD19 CAR‐T With MND Promoter Enhances Tumour Killing

**DOI:** 10.1111/jcmm.70843

**Published:** 2025-09-16

**Authors:** Xiaomei Zhang, Xiaoyuan He, Yu Zhang, Jile Liu, Shujing Guo, Cuicui Lyu, Mingfeng Zhao

**Affiliations:** ^1^ Department of Hematology Tianjin First Central Hospital, School of Medicine Tianjin China; ^2^ First Central Clinical College Tianjin Medical University Tianjin China

**Keywords:** B‐ALL, CAR‐T, CD19, MND, promoter

## Abstract

Although Chimeric antigen receptor (CAR) T cell therapy has demonstrated a high remission rate in B cell acute lymphoblastic leukaemia, concerns regarding toxicity and disease recurrence remain. Different promoters can modulate the expression levels of CAR molecules on the cell surface. In this study, we systematically compared four distinct promoters (MND, MSCV, EF‐1α and CMV). Our findings revealed that while these promoters exhibited similar characteristics, the MND promoter demonstrated superior viral packaging and transduction efficiency. Furthermore, it enhanced the anti‐leukaemia efficacy by increasing the proportion of naïve T cells involved in the cytotoxic process.

**Abbreviations**
B‐ALL: B‐cell acute lymphoblastic leukaemia
CAR‐T: Chimeric antigen receptor T cells
CMV: cytomegalovirus
CR: complete remission rate
CRS: cytokine release syndrome
DEGs: differentially expressed genes
DMEM: Dulbecco's modified Eagle medium
FACS: fluorescence‐activated cell sorting
FCS: fetal calf serum
IFN‐γ: interferon gamma
MFI: mean fluorescence intensity
MOI: multiplicity of infection
MSCV: murine stem cell virus
PBMC: Peripheral blood mononuclear cells
PEI: polyethylenimine
scFv: single‐stranded variable fragments
SD: standard deviation
TNF‐α: tumour necrosis factor alpha
UTD: untransduced T cells

## 1 Introduction

Chimeric antigen receptor T cells (CAR‐T) constitute a promising form of tumour immunotherapy that leverages genetic engineering technology to specifically recognise tumour‐associated antigens, thereby significantly enhancing the anti‐tumour efficacy [1]. In recent years, CAR‐T cell therapy has demonstrated remarkable outcomes in the treatment of leukaemia and lymphoma [2]. Multiple clinical trials utilising CD19 specific CAR‐T cell therapy have reported complete remission (CR) rates ranging from 70% to 90% in paediatric and adult patients with relapsed/refractory B‐cell acute lymphoblastic leukaemia (R/R B‐ALL) [3, 4, 5, 6]. However, its effectiveness in relapsed/refractory B‐cell lymphoma remains suboptimal, with CR rates ranging from 39% to 58% [7]. Furthermore, severe adverse effects, such as cytokine release syndrome (CRS) and neuroinflammation, which result from CAR‐T overstimulation, are common among treated patients and may lead to fatal outcomes. These findings highlight the significant need for further advancements in this therapeutic approach.

CAR structures generally consist of promoter‐driven single‐chain variable fragments (scFv), hinge regions, transmembrane domains, and one or more intracellular signalling domain structures [8]. The design of each module within the CAR framework is intricately associated with the signalling mechanism of CAR‐T cells, influencing their ultimate function and potential toxicity [9]. Although numerous studies have concentrated on optimising the scFv, hinge region, transmembrane domain, and cytoplasmic signalling domains of CARs to enhance the functionality of CAR‐T cells [8, 9], the crucial role of the promoter in driving these structural components has been largely underappreciated [10].

Preliminary studies have demonstrated that promoters play a critical role in achieving optimal expression levels of CAR genes in CAR‐T cells for the production of functional proteins [11]. Overexpression of CAR can lead to antigen‐independent signalling, resulting in T cell exhaustion and suboptimal anti‐tumour responses, or cause inappropriate recognition of tumour antigen on self‐tissue [12, 13]. Furthermore, appropriate CAR expression is beneficial for the formation of memory T cells [14]. Based on these findings, we hypothesise that the choice of promoter may significantly influence the efficacy of CAR‐T cells by modulating CAR transduction levels, surface density, and cell type specificity.

In current CAR‐T cell research, several promoters including MSCV (murine stem cell virus), CMV (cytomegalovirus), EF‐1α (elongation factor‐1 alpha), as well as MND (myeloproliferative sarcoma virus MPSV enhancer, negative control region NCR deletion, d1587rev primer binding site replacement) have been widely employed [12, 15, 16, 17, 18, 19, 20]. To systematically evaluate promoter performance in lentiviral CAR vectors, we conducted a comparative analysis of these four clinically relevant regulatory elements. Our investigation focused on their capacity to drive CAR protein expression and modulate the functional characteristics of CD19 CAR‐T cells. Comprehensive assessments included quantitative protein expression profiling, in vitro cytolytic activity measurements, and multiparametric functional characterisation. While all promoters generated CAR‐T products with comparable baseline attributes, including CD4/CD8 subset distribution, exhaustion markers (PD‐1, LAG‐3 and TIM‐3), and proliferation kinetics—the MND promoter exhibited higher levels of functional protein expression while enhancing target cell killing without increasing cytokine secretion.

## 2 Materials and Methods

### 2.1 Lentivirus Production

**FIGURE 1:**
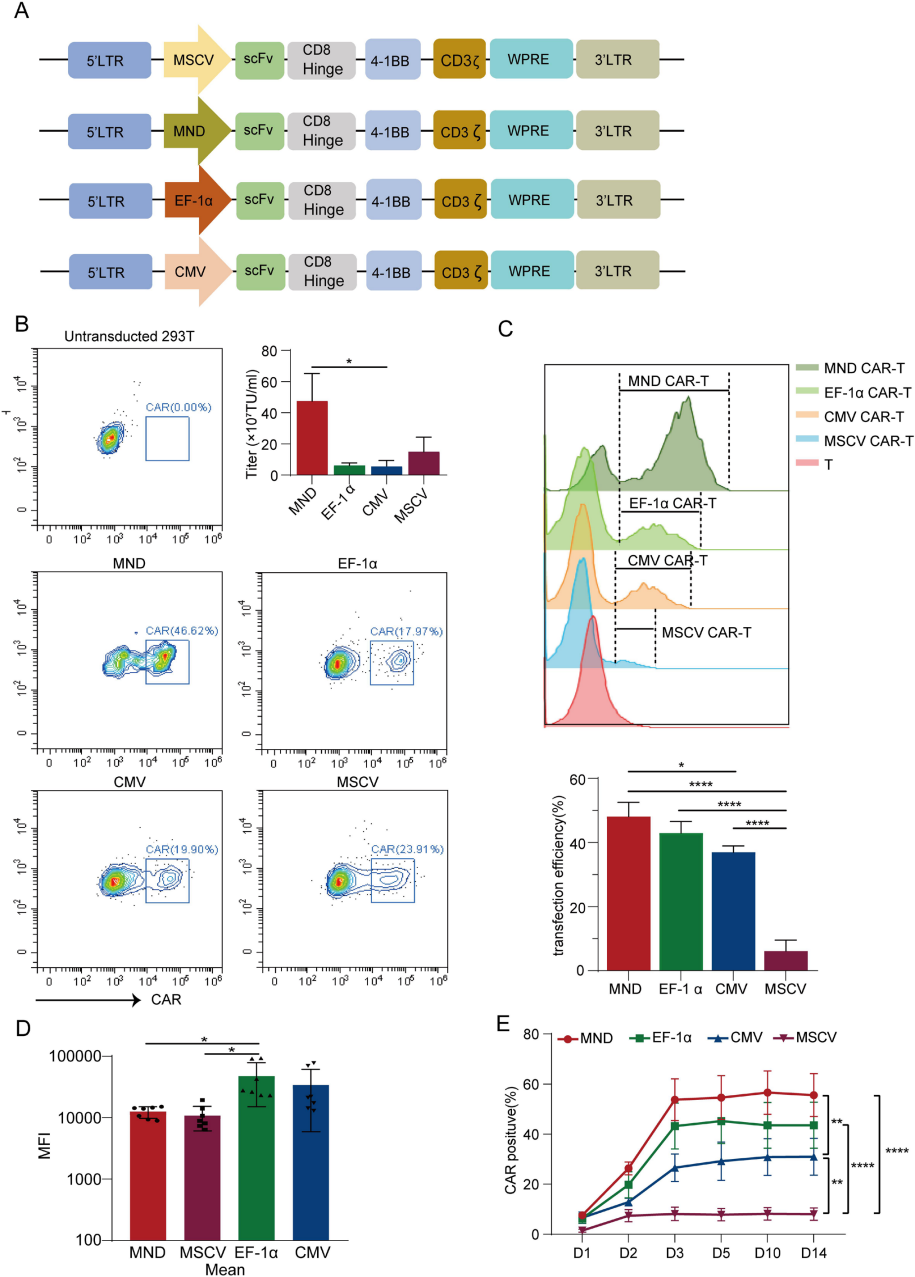
Construction of CAR‐T cells using diverse promoters and assessment of titre and transduction efficiency. (A) Construction of CD19 CAR sequences driven by various promoters (MND, MSCV, EF‐1α and CMV) was undertaken. The structure is identical except for the promoter. (B) Quantification of CD19 CAR viral titers under the influence of distinct promoters. Shown are data from three independent experiments from three donors. *p = 0.0486. (C) Transduction rate of CD19 CAR‐T cells driven by different promoters (MOI = 5). Shown are data from three independent experiments from three donors. *p = 0.0191, ****p < 0.0001. (D) MFI values of CD19 CAR‐T cells under the control of different promoters. Shown are data from seven independent experiments from four donors. (E) Temporal variations in transduction efficiency of CAR‐T cells harbouring distinct CD19 promoters following transduction. Shown are data from three independent experiments from three donors. MND vs. CMV, **p = 0.0022; CMV vs. MSCV, **p = 0.0064; ****p < 0.0001.

Figure 1A illustrated a schematic diagram of the CAR used in this study. All CARs, except for the promoter, contain the same CD19 scFv sequence and 4‐1BB co‐stimulatory domain. The preparation of the lentivirus was performed according to the manufacturer's instructions (GeneCopoeia, Beijing, China). Two days before transduction, HEK293T cells were plated in a 10 cm dish in 10 mL of DMEM supplemented with 10% heat‐inactivated fetal bovine serum so that the cells are 70% to 80% confluent at the moment of transduction. 10 μg of lentiviral vector with the appropriate insert, 10 μg of psPAX2 and 5 μg pMD2.G were cotransduced into HEK293T cells using the polyethylenimine (PEI) method. 24 h after transduction, the medium was replaced with fresh medium containing 10% heat‐inactivated fetal bovine serum. Culture supernatant was collected 48 to 72 h after transduction, and the supernatant was cryo centrifuged at 500 g for 16 min at 4°C and filtered through a 0.45 μm SFCA syringe filter. The filtered virus was stored at −80°C until use.

### 2.2 Determination of Lentiviral Titre

The supernatant was collected for p24 determination using ELISA (#631476, Takara). HEK293T cells were seeded 12 h before transduction. Then, serial dilutions of virus were transduced on top of the cells in complete DMEM + 5 μg/mL Polybrene. 72 h after transduction, the cells were trypsinised and terminated with medium. The CAR was labelled with anti‐FMC63 antibody (#FM3‐HPY53, Acro). This antibody has been previously utilised in our laboratory for similar applications [21]. The percentage of CAR positive cells was determined by fluorescence‐activated cell sorting (FACS) analysis. The titre of the lentiviral vector ranges from 10^6^ to 10^7^ transduction units per millilitre (TU/mL), which is calculated as the number of CAR positive cells divided by the dilution factor.

### 2.3 Cell Lines and Primary Cells

HEK293T(ATCC) cells were grown in Dulbecco's modified Eagle medium (DMEM; Gibco) supplemented with 10% fetal calf serum (FCS; Biological Industries). Nalm6, U2932 and Jurkat cells were grown in RPMI 1640 (Invitrogen). All cell lines were cultured at 37°C, 5% CO_2_ and 95% humidity. The cells were divided every 2 to 3 days, and the number of passages did not exceed 20. Peripheral blood samples were obtained from healthy donors in Tianjin First Central Hospital after informed consent was obtained according to the institutional guidelines. Peripheral blood mononuclear cells (PBMCs) were enriched through a Ficoll Hypaque gradient. Human CD3 T cells were isolated using CD3 immunomagnetic beads (#130‐097‐043, Miltenyi Biotec, Germany). T cells were amplified using CD3/CD28 stimulation beads (#11131D; Thermo Fisher Scientific, USA) and IL‐2 (250 U/mL; Miltenyi Biotec, Germany) in X‐VIVO 15 Cell Medium (Lonza, Switzerland).

### 2.4 Lentiviral Transduction of Primary Human T Cell

Primary human T cells were activated and expanded for 48 h, followed by being transduced with lentivirus (multiplicity of infection is five) incubation in the presence of polybrene (Sigma, USA) at 5 μg/mL. Subsequently, the cells were continued to expand at 37°C and maintained at an appropriate concentration (0.5–1 × 10^6^ cells/mL). The transduction efficiency was determined 3 days after transduction. Generally, the T cells were engineered via 9–12 days of manufacturing to express a different promoter CD19‐specific CAR.

### 2.5 Animal Model of B‐ALL and Study Design

Male NSG mice (8–12 weeks old; 18–22 g, Sbefer, Beijing, China) were housed in SPF cages with free access to standard food and water.

Mice were grouped as indicated in the experiments described below, each group consisting of three mice. All mice were injected intravenously with 2 × 10^6^ tumour cells expressing luciferase. Then these mice were randomly divided into five groups. After 3 days, 5 × 10^6^ CAR positive T cells or uninfected T cells were injected into the mice through the tail vein. To monitor tumour growth, each murine was intraperitoneally injected with 3 mg of D‐fluorescein (Sigma) at the designated time point. Ten minutes later, the mice were imaged with an IVIS Lumina II (PerkinElmer, USA) to assess tumour burden. At the same time, the expansion level and subpopulation distribution level of CAR‐T cells in mice were detected by tail vein blood collection. According to the requirement of the Animal Ethics Committee, the mice were sacrificed once they developed hind‐limb paralysis or lost 20% of their body weight.

### 2.6 Cell Counting Kit (CCK‐8) Assay

Cells (5 × 10^4^/100 μL/well) were inoculated in 96‐well plates and incubated for 24 h (37°C, 5% CO_2_). Added 10 μL of CCK‐8 solution (#C0048M, Beyotime, China) to each well, and incubated for another 2 h. Optical density was measured at 450 nm using an enzyme marker (Thermo, USA).

### 2.7 CAR‐T Cell Toxicity Assay

The cytotoxicity of CAR‐T cells was performed by the bioluminescence assay. Briefly, 1 × 10^5^ Nalm6‐GFP‐Luc cells were cultured in a 24‐well microplate with RPMI 1640 (Invitrogen) medium in the presence of different ratios of transduced CAR‐T cells at 37°C in 5% CO_2_ for different times. The percentage of tumour cell viability was calculated based on flow cytometry compared with tumour cells alone. All data were expressed as mean ± SD of three independent experiments with triplicate wells each.

### 2.8 CAR‐T Cell Subpopulation Detection

CAR‐T cells were immunophenotyped using the following antibody panel: anti‐CD3 antibody (#130‐115‐972; Miltenyi Biotec, Germany) for general T cell identification, anti‐FMC63 antibody for CAR detection, anti‐CD4 (#130‐115‐434; Miltenyi Biotec) and anti‐CD8 (#130‐115‐197; Miltenyi Biotec) antibodies for T cell subset differentiation. To assess T cell differentiation status, we employed the panel containing anti‐human CD45RA PE‐Vio770 and anti‐human CD62L APC surface markers. Furthermore, exhaustion markers on CAR‐T cells were analysed through triple staining with anti‐PD‐1 (#329920; Biolegend), anti‐TIM3 (#345028; Biolegend) and anti‐CTLA4 (#369624; Biolegend) antibodies.

### 2.9 Bulk RNA‐Seq

Different promoter CAR‐T cells constructed from T cells derived from healthy donors were collected 8 days after transduction. Using anti‐FMC63 antibody and anti‐PE antibody for magnetic bead sorting, a total of 5 × 10^6^ CAR positive T cells were obtained for transcriptome sequencing. The raw data was obtained using the Illumina sequencing platform provided by Novogene. The transcriptomic sequencing results were analysed using RSEM software. Fold Change ≥ 2 and FDR < 0.01 were used as differential expression gene screening criteria.

### 2.10 Statistical Analysis

Data were expressed as mean ± standard deviation (SD) for in vitro studies, and as mean ± standard error of the mean (SEM) for in vivo study. All data analyses were performed by unpaired t‐test or one way/two way analyses of variance (ANOVA) followed by post hoc Tukey's multiple comparison test or Sidak's multiple comparisons test. *p* < 0.05 was considered to be statistically significant. All statistical data were analysed with Graphpad Prism 8 software (Graphpad Software Inc., CA).

## 3 Result

### 3.1 Comparison of Vector Expression in Redirected and Primary T Cells

Different internal promoters were selected to construct lentiviral vectors for optimising gene expression in primary T cells (Figure 1A), with the primary consideration being their impact on viral titre and transduction efficiency. We investigated whether the choice of different internal promoters would affect viral titre and transduction in primary T cells, as shown in Figure 1B; Figure S1. By using a dilution method and ELISA to measure infectious and physical viral titres after transducing HEK293T cells with CAR‐labelled lentivirus, we observed varying levels of viral titres among HEK293T cells transduced with different promoter‐driven viruses. Among them, MND virus exhibited higher viral concentration compared to the other three promoters. Exposing each vector to an equal multiplicity of infection (MOI = 5) and analysing CAR expression through flow cytometry after 3 days, we found that all lentiviral vectors were capable of transducing T cells. The MND promoter showed the highest level of CAR expression, while MSCV‐mediated CAR transduction had the lowest efficiency (Figure 1C). Despite the MND promoter achieving the highest transduction efficiency in T cells, mean fluorescence intensity (MFI) analysis revealed that the surface expression level of CAR in T cells driven by the MND and MSCV promoters was lower compared to that driven by the EF‐1α promoter post‐manufacture (Figure 1D). We tracked the transduction efficiency, finding that all the four promoters increased exactly within the 72‐h mark and became stable after a few days. The transduction efficiency of MND CAR‐T cells is significantly higher than CMV CAR‐T cells and MSCV CAR‐T cells. Although the transduction rate of MND CAR‐T was higher than that of EF‐1α CAR‐T, the difference was not statistically significant (Figure 1E).

### 3.2 CAR‐T Cell Subsets With Different Promoter at Baseline

**FIGURE 2:**
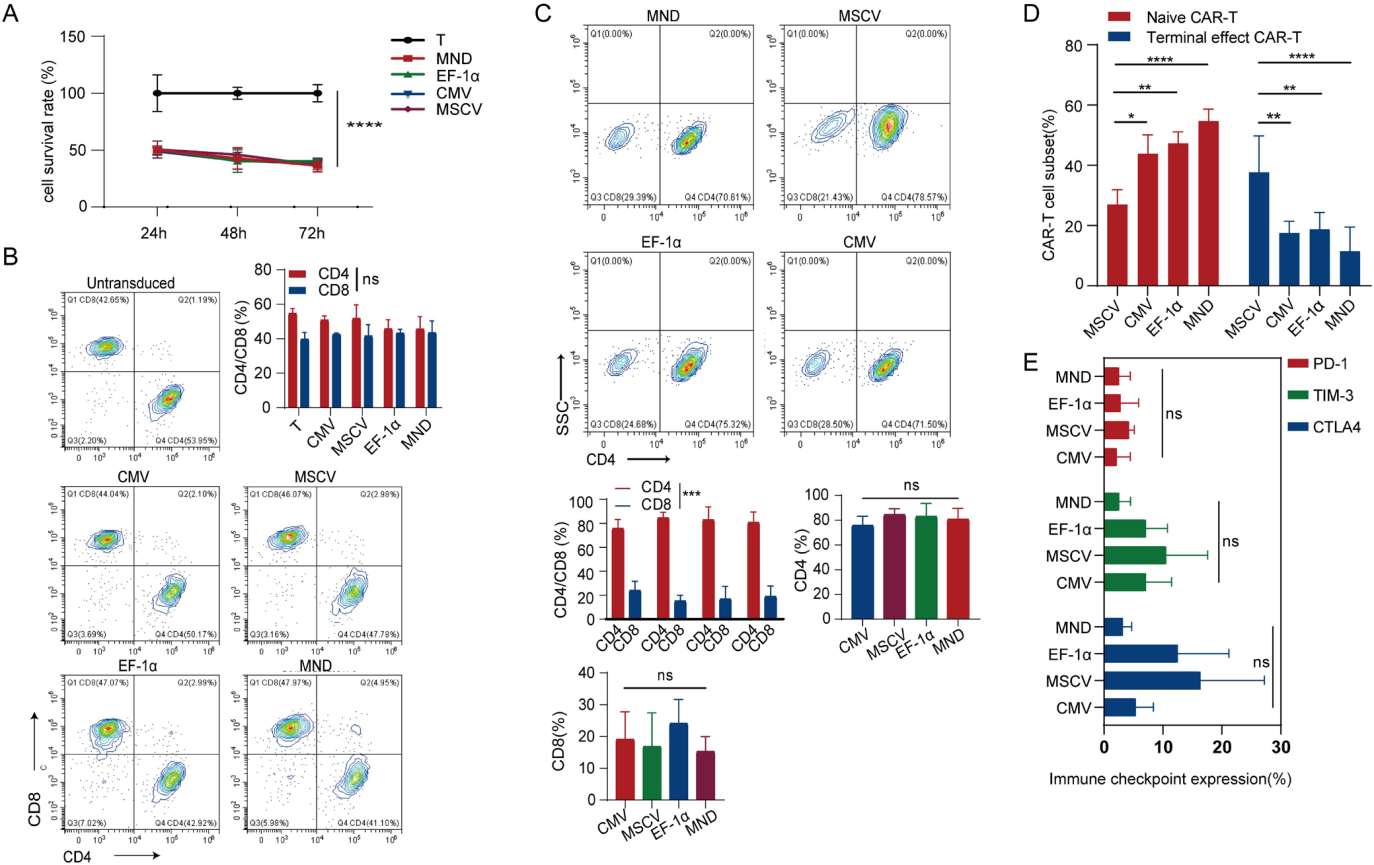
Comparison of proliferation capacity and baseline subpopulation ratio of, as well as the exhausted markers of CAR‐T cells utilising different promoters. (A) Proliferation of CAR‐T cells driven by different promoters within 3 days after transduction. Shown are data from three independent experiments from three donors. ****p < 0.0001. (B, C) Disparities in CD4 and CD8 ratios between CD19 CAR‐T cells (B) and CAR positive T cells (C) are influenced by distinct promoters. Shown are data from three independent experiments from three donors. ***p < 0.01. The proportions of baseline subpopulations (D) and the expression of immune checkpoint (E) in CD19 CAR‐T cells with different promoters were compared. Shown are data from three independent experiments from three donors.

In order to clarify whether different promoters would interfere with the functional status of CAR‐T cells transduced by them, we first examined their baseline proliferation, subpopulations, and checkpoint expression. The results showed no significant differences in proliferation among CAR‐T cells driven by different promoters, and all were weaker than the proliferation state of untransduced T cells (UTD) (Figure 2A). Next, we assessed the functionality of each promoter construct‐transduced CAR‐T cells by measuring subpopulations and exhaustion marker expression before conducting toxicity assays. Although CAR‐T cells with different promoters had similar ratios of CD4 and CD8 compared to UTD when mixed with CAR negative T cells (Figure 2B), separating out the CAR positive T cells revealed a significantly higher proportion of CD4 + CAR‐T cells compared to the UTD group. However, there was no significant difference in the ratio of CD4 cells to CD8 cells among four groups of CAR positive T cells driven by different promoters (Figure 2C). Furthermore, when examining CAR positive T cells with different promoters, it was found that the MSCV promoter‐driven CAR positive naïve T cell population (CD45RA + CD62L+) was lower than that in the other three promoter‐driven CAR‐T cell populations while the effector T cell population (CD45RA + CD62L−) was higher than that in the other three promoter‐driven CAR‐T cell populations (Figure 2D). There were no differences observed in exhaustion marker expression between four types of promoter‐driven CAR‐T cell populations (Figure 2E).

### 3.3 MND Drives CAR‐T Cells to Effectively Kill CD19 Cells While Maintaining Stem Cell‐Like T Cell Subset

**FIGURE 3:**
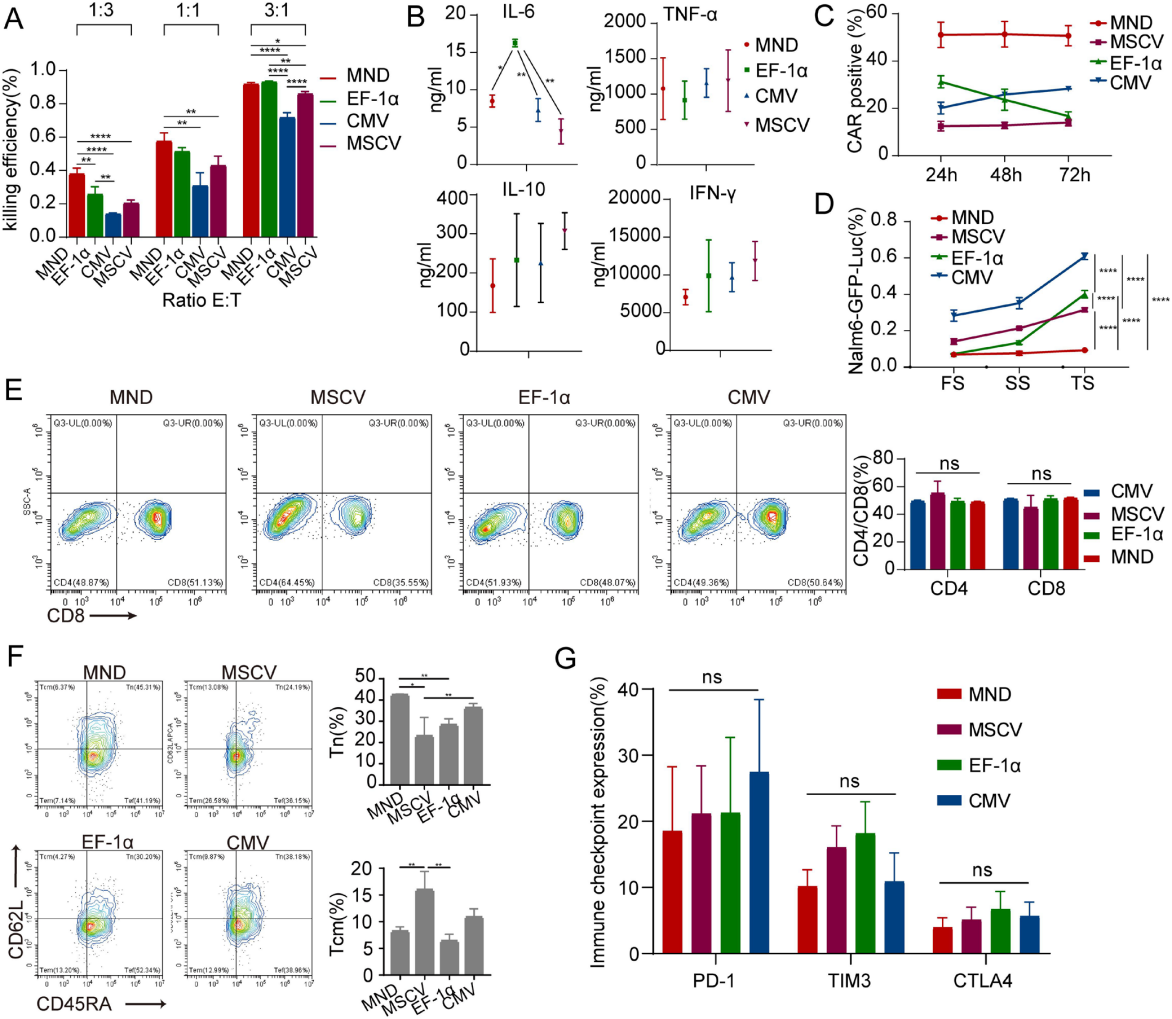
Tumour lysis efficacy and phenotypic variation of CAR‐T cells with distinct promoters following cytotoxicity. (A) Comparing the killing effect of CD19 CAR‐T cells driven by different promoters under different effect‐target ratios. (E:T 1:3, 1:1, 3:1). Shown are data from three independent experiments from three donors. (E:T 1:3) MND vs. EF‐1α, *p* = 0.0051; MND vs. CMV, *p* < 0.0001; MND vs. MSCV, *p* = 0.0005; EF‐1α vs. CMV, *p* = 0.0069. (E:T 1:1) MND vs. CMV, *p* = 0.0017; EF‐1α vs. CMV, *p* = 0.0089. (E:T 3:1) MND vs. CMV, *p* < 0.0001; MND vs. MSCV, *p* = 0.0187; EF‐1α vs. CMV, *p* < 0.0001; EF‐1α vs. MSCV, 0.0061; CMV vs. MSCV, *p* < 0.0001. (B) The expression of cytokines was compared among CAR‐T cells driven by different promoters following 48 h of targeted cytotoxicity. Shown are data from three independent experiments from three donors. IL‐6, MND vs. EF‐1α, *p = 0.0109; EF‐1α vs. CMV, **p = 0.0064; EF‐1α vs. MSCV, **p = 0.0022. (C) Modulation of CAR positive T cell expression in CAR‐T cells under the influence of different promoters during cytotoxicity. Shown are data from six independent experiments from six donors. (D) The same number of Nalm6‐GFP‐Luc cell line was added into the four CAR‐T cell systems at 1:1 efficacy ratio, and after detected to be complete, the same number of cell line was added again. Shown are data from three independent experiments from three donors. ****p < 0.0001. FS, first stimulus; SS, second stimulus; TS, third stimulus. (E–G) The proportions of T cell subpopulations (E, F) and the expression of immune checkpoint (G) after killing in CD19 CAR‐T cells with different promoters were compared. Shown are data from three independent experiments from three donors.

Part of the loss of CAR‐T efficacy comes from early failure of CAR‐T cells due to excessive CAR signalling after antigen exposure. Overstimulation of CAR‐T cells also produces CRS, which is promoted by increased release of pro‐inflammatory cytokines. We therefore evaluated the cytotoxic potential, tumour necrosis factor alpha (TNF‐α), interferon gamma (IFN‐γ), and IL‐6 secretion, phenotype, and depletion markers of four promoter CAR‐T cells. One CD19 tumour model, Nalm6‐GFP‐Luc (derived from BALL), was incubated with four CAR‐T cells, and different parameters were analysed, as shown in Figure 3, where MND‐driven CAR‐T cells showed stronger anti‐tumour activity than MSCV CAR‐T cells and CMV CAR‐T cells (Figure 3A). To evaluate the cytotoxicity against antigen‐low target cells, we additionally seeded U2932 (low CD19 expression) and Jurkat (no CD19 expression) cells alongside Nalm6 cells. Our results demonstrate that the four groups of promoter‐driven CAR‐T cells exhibited diminished tumour‐targeting efficacy when tumour surface antigen density was reduced; however, MND CAR‐T cells maintained the highest cytotoxic activity (Figure S2A,B). Moreover, the secreted cytokine levels were relatively low, including TNF‐α, IFN‐γ, IL‐6 and IL‐10 (Figure 3B). After receiving the tumour rechallenge assay, it can still maintain the strong tumour killing ability of the MND CAR‐T cell, showing its strong anti‐tumour persistence (Figure 3D). The transduction rate of CAR‐T cells with different promoters was monitored during the killing process, and it was found that the transduction rate of EF‐1α CAR‐T cells gradually decreased in the late killing period (Figure 3C). Interestingly, during the killing process, the proportion of CD8+ cells in CAR positive T cells with different promoters increased significantly, but there was no difference in the proportion of CD8 + CAR‐T cells among promoters (Figure 3E). We also observed that MND CAR‐T cells and MSCV CAR‐T cells retained a poorly differentiated phenotype after killing Nalm6‐GFP‐Luc cells (Tn, CD45RA + CD62L+; Tcm, CD45RA‐CD62L+) (Figure 3F). Interestingly, the expression of depletion markers in MND CAR‐T cells after killing Nalm6‐GFP‐Luc showed a downward trend compared with other promoter‐driven CAR‐T cells, but the difference was not statistically significant (Figure 3G).

### 3.4 Nalm6 Modelling With NSG Mice Demonstrated That MND CAR‐T Cells Have Better In Vivo Anti‐Tumour Efficacy

**FIGURE 4:**
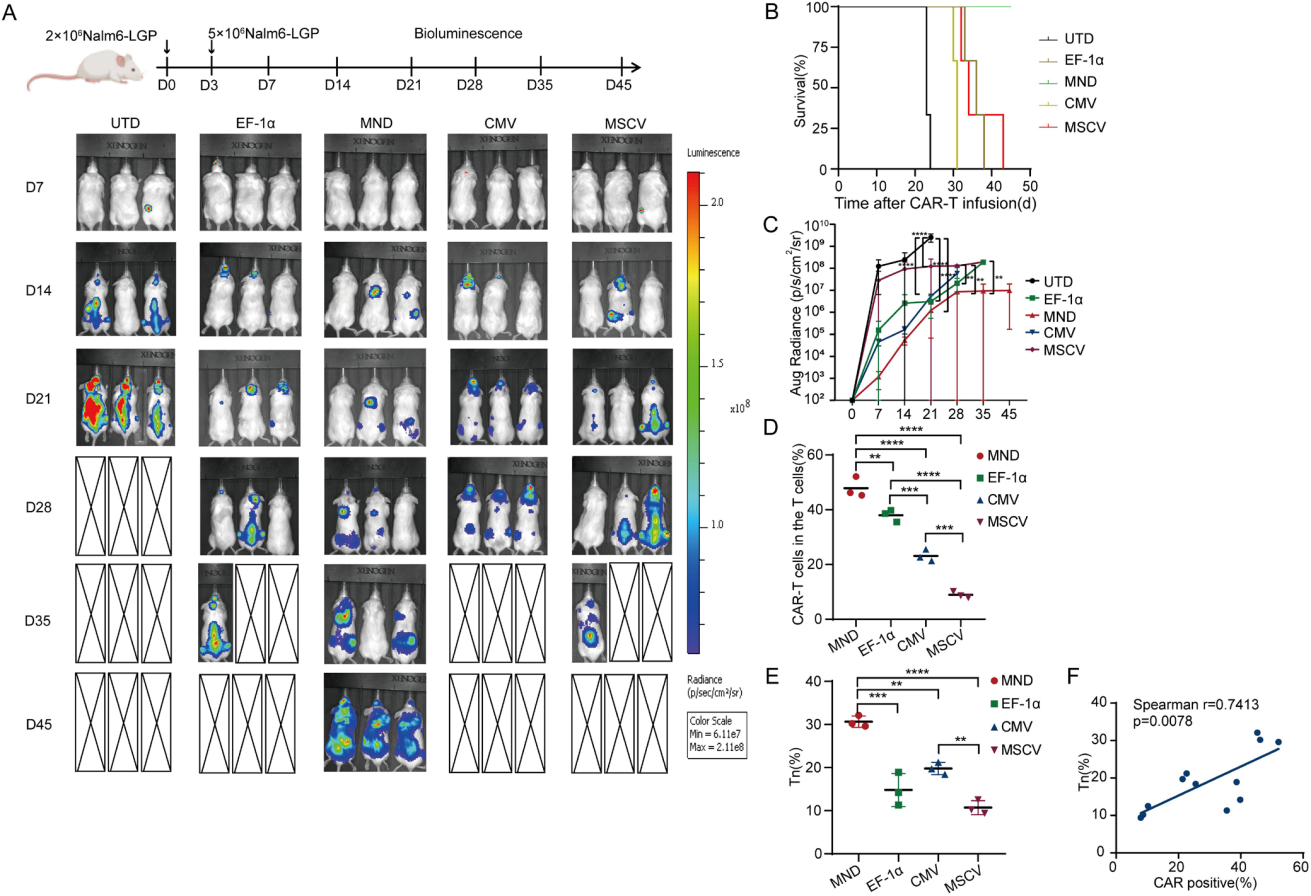
In vivo comparison of the anti‐leukaemia effects of CAR‐T cells utilising distinct promoters. (A) In vivo experimental design of the tumour engraftment, CAR‐T cell treatment, and observation timeline. First 2 × 10^6^ Nalm6‐GFP‐Luc cells were infused into NSG mice. The mouse model demonstrated the antileukemia effect of different promoter CAR‐T cells by BLI of the transplanted mice (*n* = 3). (B, C) The survival time and tumour load of mouse with different promoter CAR‐T cells were compared; *p < 0.05, **p < 0.01, ***p < 0.001. (D) Flow cytometry was used to detect the proliferation of CAR‐T cells in mice (*n* = 3). (E) Flow cytometry was used to detect the subpopulation of CAR‐T cells in mice (*n* = 3). (F) Spearman correlation coefficient was used to analyse the correlation between Tn expression level and CAR‐T cell proliferation (spearman *r* = 0.7413, *p* = 0.0078).

To compare the anti‐leukaemia activity of four promoter‐driven CAR‐T cells, 5 × 10^6^ CAR‐T cells were intravenously injected into each mouse (Figure 4A). In the control group, all mice died on day 23 and 24 respectively. CMV CAR‐T group mice died on day 30 and 31, EF‐1α CAR‐T group mice died on day 33, 36, and 38, MSCV CAR‐T group mice died on day 32, 34, and 43. On observation until day 45, MND‐CAR T group mice remained alive (Figure 4B). Bioluminescence imaging (BLI) results showed that tumour cell proliferation in mice treated with MND‐CAR T cells was initially significantly inhibited (Figure 4C). We collected venous blood from mice every week to detect the level of CAR‐T cells in mice, finding that MND CAR‐T cells had the highest level in vivo at the peak of expansion, followed by EF‐1α CAR‐T cells, and MSCV CAR‐T cells had the lowest level in vivo at the peak of expansion (Figure 4D). Interestingly, at the same time, the distribution of CAR‐T cell subsets was detected, and it was found that the proportion of Tn subsets in MND CAR‐T cells was the highest (Figure 4E), and there was a significant positive correlation between Tn subpopulation and CAR positive cells (Figure 4F).

### 3.5 RNA‐Seq in Different Promoter CAR‐T Cells

#### 3.5.1 Analysis of Differentially Expressed Genes

**FIGURE 5:**
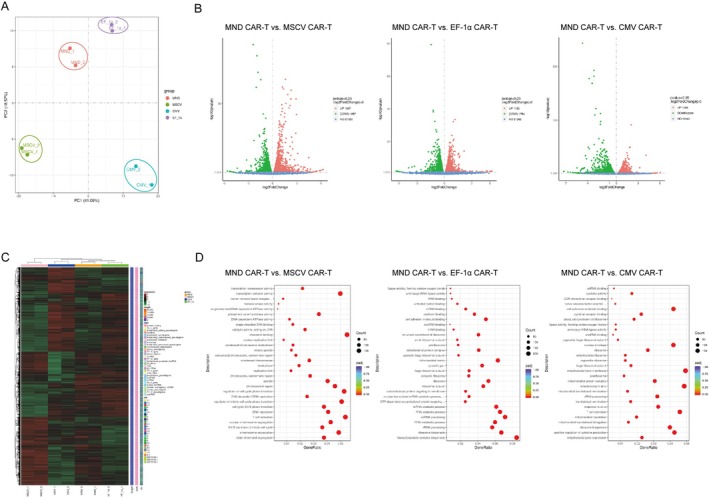
Transcriptomic disparities among CAR‐T cells employing distinct promoters. (A) PCA analysis of CAR‐T cells driven by different promoters. (B) Differential gene volcano between MND promoter driven CAR‐T cells and other promoter driven CAR‐T cells. (C) Heatmap of differential gene expression in CD19 CAR‐T cells by different promoters. (D) GO enrichment pathway analysis of CD19 CAR‐T cells driven by MND promoter and other CAR‐T cells driven by other promoters.

In order to understand the differences in gene expression of CAR‐T cells driven by different promoters, we collected RNA‐seq data from CAR positive T cells stimulated for 8 days prior to anti‐tumour response, aiming to explore transcriptional variations. Principal component analysis (PCA) showed that the four groups were clearly distinguishable from each other (Figure 5A). We found a total of 3694 differentially expressed genes (DEGs) between MND CAR‐T and MSCV CAR‐T groups, with 1997 genes upregulated and 1697 genes downregulated in the MND CAR‐T group. There existed a total of 3236 DEGs between MND CAR‐T and EF‐1α CAR‐T groups, with 1452 genes upregulated and 1784 genes downregulated in the MND CAR‐T group. Similarly, in the comparison between MND CAR‐T and CMV CAR‐T groups, we identified a total of 4215 DEGs, with 1926 genes upregulated and 2289 genes downregulated in the MND CAR‐T group (Figure 5B), and Heatmap analysis showed that the differential genes of the four groups had obvious differentiation (Figure 5C).

#### 3.5.2 Enrichment Analysis

We performed GO enrichment analysis on the aforementioned DEGs. The results of GOBP and GOMF enrichment analysis showed that the differentially expressed genes between MND CAR‐T and MSCV CAR‐T were mainly enriched in biological functions such as ‘T cell activation’, ‘DNA replication’, ‘transcriptional activity’, and ‘cell cycle’, as well as molecular functions categories such as ‘histone kinase activity’, ‘tumour necrosis factor receptor superfamily binding’ and ‘cytokine receptor binding’. The differentially expressed genes between MND CAR‐T and EF‐1α CAR‐T were also enriched in biological functions including ‘transcriptional activity’, ‘lymphocyte activation’, ‘T cell activation’, ‘cell adhesion’. The differentially expressed genes between MND CAR‐T and CMV CAR‐T were mainly enriched in biological functions like ‘positive regulation of cytokine production’, ‘T cell activation’, ‘regulation of innate immune response’, ‘response to interferon‐γ’, ‘interferon‐γ‐mediated signaling pathway’, ‘immune response‐activating signal transduction’, along with molecular functions like ‘cytokine receptor binding’, ‘tumour necrosis factor receptor binding’ (Figure 5D). These function states indicate that MND CAR‐T cells are in a highly activated and enhanced immune response state, and exhibit significant biological functions in immune response activation signal transduction, enhancing the anti‐tumour immune response.

KEGG enrichment analysis of MND CAR‐T and MSCV CAR‐T revealed enrichment in ‘DNA replication’, ‘cell cycle’, ‘apoptosis’, ‘NOD‐like receptor signaling pathway’, ‘p53 signaling pathway’, ‘FOXO signaling pathway’, and ‘necroptosis’ (Figure S3A). The KEGG analysis of MND CAR‐T and EF‐1α CAR‐T reveals that KEGG is mainly enriched in ‘JAK‐STAT signaling pathway’, ‘antigen processing and presentation’, ‘ribosome’, ‘apoptosis’, ‘T‐cell receptor signaling pathway’, ‘RNA transport’, ‘cell cycle’, and ‘DNA replication’ (Figure S3B). When comparing the MND CAR‐T and CMV CAR‐T, it can be observed that there are interactions between ‘cytokines and cytokine receptor’, ‘antigen processing and presentation’, ‘TNF signaling pathway, apoptosis’, ‘p53 signalling pathway’, ‘NOD‐like receptor signalling pathway’, ‘TH17 cell differentiation’, and ‘IL17 signaling pathway’ (Figure S3C). The above enrichment results indicate that CAR‐T cells are in a state where active proliferation and stress regulation coexist, suggesting that CAR‐T cells are in an acute immune response state.

## 4 Discussion

Guedan et al. [22] investigated the effect of surface CAR expression on CAR driven by different promoters. Another study compared four promoter lentiviral titers, transduction efficiency, marker and CAR expression levels, cytokine production, and killing capacity. EF‐1α showed the best transduction efficiency, killing capacity, and cytokine production [11]. In our study, we found that EF‐1α had better packaging efficiency and transduction efficiency than CMV. But the MND promoter was observed to enhance packaging efficiency obviously rather than EF‐1α, although they have the same transduction efficiency. Ho et al. [23] propose that the density of chimeric antigen receptors (CARs) on T cell surfaces can regulate both efficacy and safety. They also suggest that employing the MND promoter can enhance transduction efficiency while reducing CAR surface density. Furthermore, they demonstrate that decreasing CAR molecular surface density does not compromise the anti‐tumour effect in vivo or in vitro; however, it may result in cytokine reduction.

In our observation, it was found that MND CAR‐T cells not only had strong packaging efficiency, but also showed the best transduction efficiency and killing ability, with moderate MFI expression of transduction rate, as well as moderate cytokine secretion ability. A previous study has shown that high expression of CAR in one cell may result in tetanic signal, which may induce CAR‐T cell exhaustion [23]. In our study, although MND CAR‐T cells have the highest transduction rate, the MFI of CAR in MND CAR‐T cells is not much high. This means that applying the MND promoter not only saves cell culture time, but also reduces the depletion level of CAR‐T cells.

The killing test results showed that although the early MND CAR‐T cells and EF‐1α CAR‐T cells had similar anti‐tumour effects, the tumour‐lysis ability of MND CAR‐T cells and MSCV CAR‐T cells was more durable. The analysis of T cell subsets showed that the proportion of Tn cells in MND CAR‐T cells at the late killing stage was significantly higher. The proportion of MSCV driven CAR‐T cells increased in the Tcm subpopulation, which could maintain the naive and memory‐like characteristics of CAR‐T cells. Compared with terminally differentiated Teff cells, Tn and Tcm cells possess greater potential for long‐term persistence and produce lower levels of inflammatory cytokines, which could together enable a durable anti‐tumour response while avoiding severe acute toxicities such as CRS [24, 25, 26].

Next, we compared the differential gene expression profiles between MND CAR‐T cells and other promoter‐driven CAR‐T cells through transcriptome sequencing. The results revealed that the differentially expressed genes in MND CAR‐T cells were associated with memory, proliferation, immune synapse, migration, activation, and inhibition. Furthermore, these genes were predominantly enriched in membrane composition, receptor‐mediated signalling pathways, T‐cell complexes, and involvement in TCR signalling pathways.

In summary, as a promoter, MND has demonstrated higher packaging efficiency and transduction efficiency. It also regulates CAR‐T cells to remain in a more primitive and immature stage, which may be associated with its strong cytotoxicity both in vitro and in vivo, leading to moderate secretion of cytokines. This provides evidence for its favourable therapeutic efficacy and safety. Further exploration of its mechanism suggests that the MND promoter may regulate genes related to memory during the process of driving CAR expression, thereby influencing the TCR signalling pathway and regulating T cell differentiation processes. However, further research is needed to delve into these regulatory mechanisms. The above study only applies to the CD19 target; whether the functional differences of these promoters have the same results in other target studies is unknown, and related target studies are ongoing.

## Author Contributions

**Xiaomei Zhang:** data curation (equal), formal analysis (lead), resources (lead). **Xiaoyuan He:** methodology (equal). **Yu Zhang:** data curation (equal), writing – review and editing (equal). **Jile Liu:** software (equal), writing – review and editing (equal). **Shujing Guo:** project administration (equal), validation (equal). **Cuicui Lyu:** funding acquisition (equal), project administration (equal), writing – review and editing (equal). **Mingfeng Zhao:** conceptualization (equal), funding acquisition (equal), project administration (equal), writing – review and editing (equal).

## Ethics Statement

The study was approved by the Ethics Committee of Tianjin First Central Hospital. And our study complies with the Declaration of Helsinki. Primary human CD3 + T cells were acquired from the Laboratory of Haematology Department, Tianjin First Central Hospital. Informed consent (oral and written) was obtained from all participants included in the study. All mice experiments were approved by and performed in accordance with national and regional guidelines and approved by the Animal Care and Use Committee of Tianjin Medical University (No. 2022‐SYDWLL‐000278).

## Consent

The authors have nothing to report.

## Conflicts of Interest

The authors declare no conflicts of interest.

## Supporting information


**Figures S1–S3:** jcmm70843‐sup‐0001‐Supinfo.zip.

## Data Availability

The data that support the findings of this study are available from the corresponding author upon reasonable request.
