# Supplementary material for: Targeting CD19 CAR‐T With MND Promoter Enhances Tumour Killing
Source: J Cell Mol Med. 2025 Sep 16;29(18):e70843. doi: 10.1111/jcmm.70843 (PMC12439678; doi:10.1111/jcmm.70843)
Supplement: Supplementary file 1 — Figures S1–S3: jcmm70843‐sup‐0001‐Supinfo.zip. [file JCMM-29-e70843-s001.zip › jcmm70843-sup-0004-legends.docx]

**Supplementary Fig. 1** Package efficiency of the four lentiviral vectors was determined by the concentration of HIV p24 in the supernatant. Shown are data from three independent experiments from three donors. MND vs. EF-1α, ***p=0.0002; MND vs. CMV, ***p=0.0001; EF-1α vs. MSCV, **p=0.0033; CMV vs. MSCV, **p=0.0024.

**Supplementary Fig. 2** Evaluation of cytotoxic effects exerted by CAR-T cells with diverse promoters across varying target antigen levels. A. The surface CD19 antigen-density in Nalm6, U2932, Jurkat cells; B. Comparing the killing effect of CD19 CAR-T cells driven by different promoters under different antigendensity tumor cells. Shown are data from three independent experiments from three donors. (MND) Nalm6 vs. U2932, p<0.01; Nalm6 vs. Jurkat, p<0.0001, U2932 vs. Jurkat, p<0.0001. (EF-1α) Nalm6 vs. U2932, p <0.0001; Nalm6 vs. Jurkat, p<0.0001, U2932 vs. Jurkat, p<0.0001. (CMV) Nalm6 vs. U2932, p<0.05; Nalm6 vs. Jurkat, p<0.0001, U2932 vs. Jurkat, p<0.0001. (MSCV) Nalm6 vs. U2932, p<0.0001; Nalm6 vs. Jurkat, p<0.0001, U2932 vs. Jurkat, p<0.0001. (Nalm6) MND vs. CMV, p<0.01; EF-1α vs. CMV, p<0.01; CMV vs. MSCV, p<0.05; (U2932) MND vs. EF-1α, p<0.05. MND vs. CMV, p<0.0001; MND vs. MSCV, p < 0.001; EF-1α vs. MSCV, p<0.01.

**Supplementary Fig. 3** KEGG enrichment pathway analysis of CD19 CAR-T cells driven by MND promoter and other CAR-T cells driven by other promoters.
