# Supplementary figures and images for: Targeting CD19 CAR‐T With MND Promoter Enhances Tumour Killing
Source: J Cell Mol Med. 2025 Sep 16;29(18):e70843. doi: 10.1111/jcmm.70843 (PMC12439678; doi:10.1111/jcmm.70843)

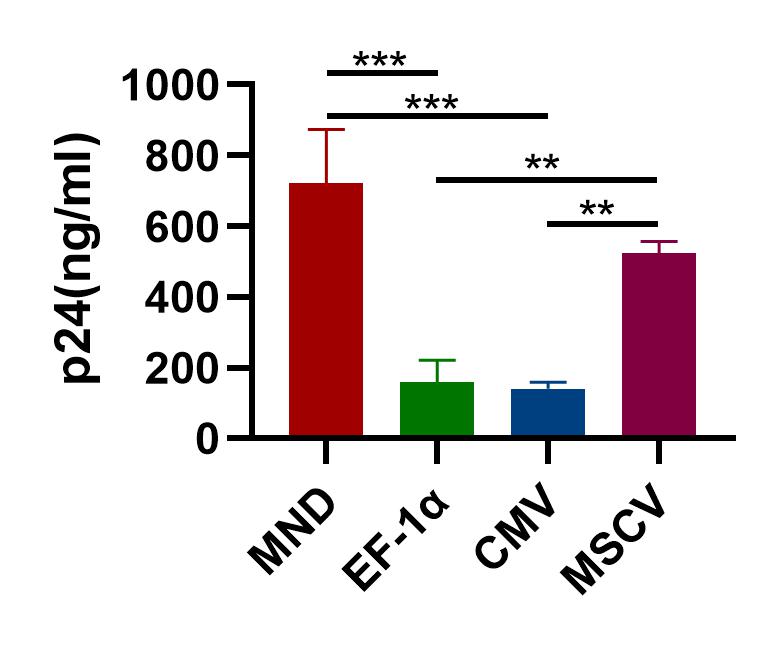

Supplement: Supplementary file 1 — Figures S1–S3: jcmm70843‐sup‐0001‐Supinfo.zip. [file JCMM-29-e70843-s001.zip › jcmm70843-sup-0001-FigureS1.jpg]

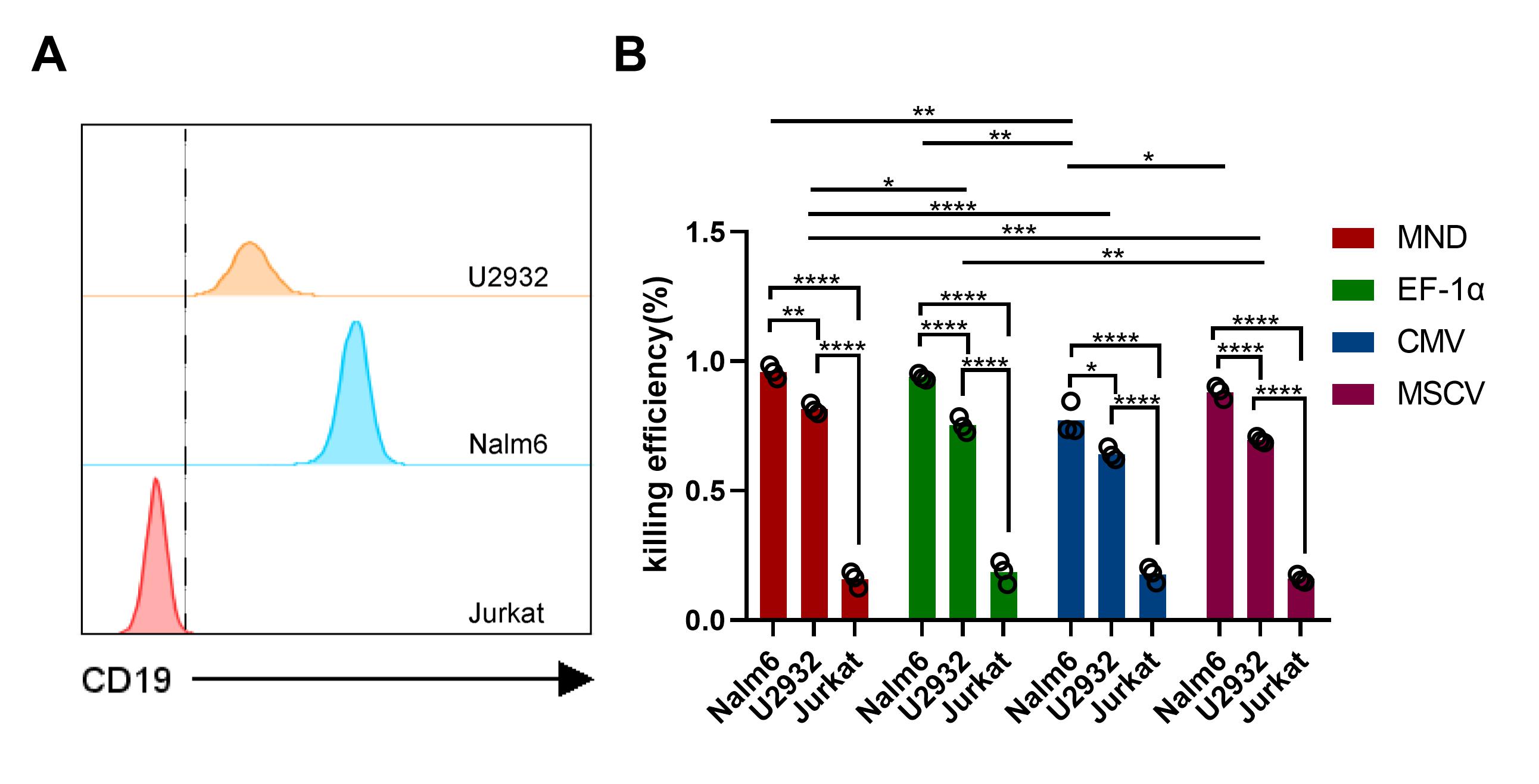

Supplement: Supplementary file 1 — Figures S1–S3: jcmm70843‐sup‐0001‐Supinfo.zip. [file JCMM-29-e70843-s001.zip › jcmm70843-sup-0002-FigureS2.jpg]

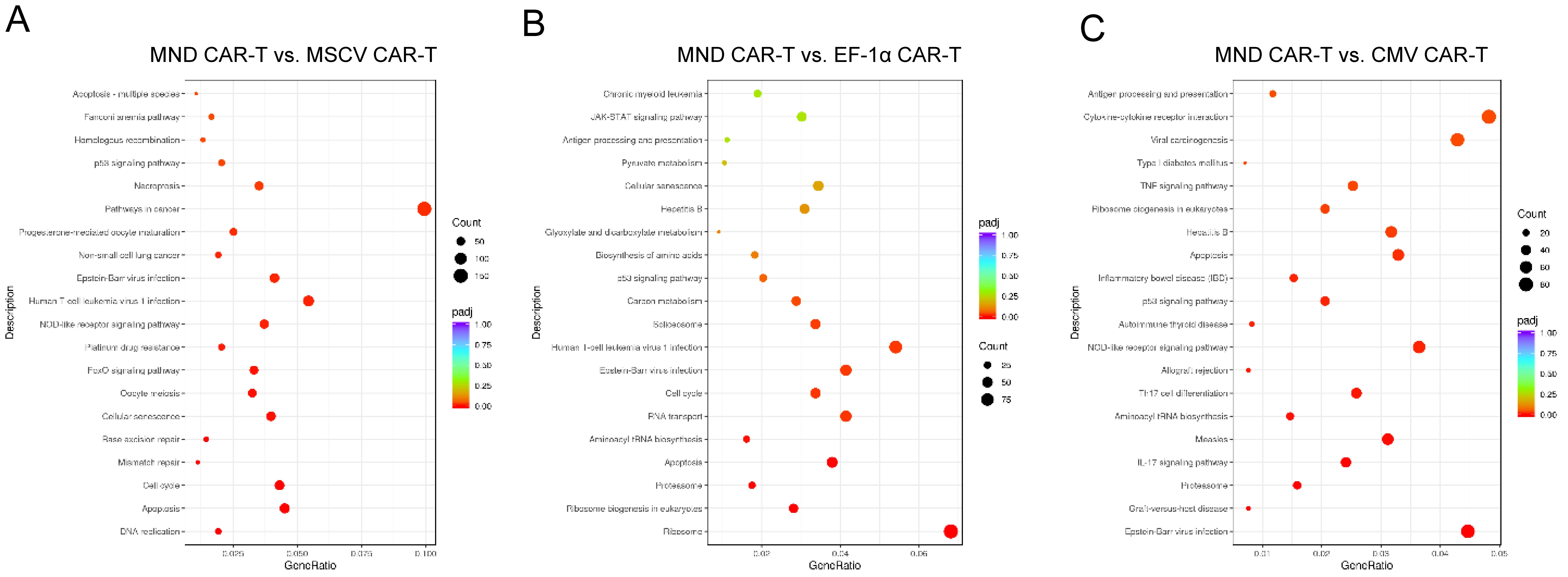

Supplement: Supplementary file 1 — Figures S1–S3: jcmm70843‐sup‐0001‐Supinfo.zip. [file JCMM-29-e70843-s001.zip › jcmm70843-sup-0003-FigureS3.jpg]
